# The Epileptic Colony at Chalfont

**Published:** 1907-01-19

**Authors:** 


### THE EPILEPTIC COLONY AT CHALFONT.

It is only in comparatively recent times that anything practical has been done for epileptics. These unfortunate creatures were classed with the insane, although between their attacks they were perfectly reasonable. But liable as they are to be at any time disabled by a fit, they cannot follow regular occupations under ordinary conditions, nor is every occupation suited for them. Outdoor work is the best for them, and to provide them with this the Chalfont Colony for Epileptics was founded in 1894. At first only eighteen invalids were received, but the work has grown, as its value has been realised, and the colony is now a little industrial village with a population of 200 patients. So far the place has been managed without running into debt, but there is now a need for more land to give occupation to the invalids, and when it happened that a farm of 150 acres in the neighbourhood of the colony was for sale at the price of £5,500, which is less than land in the neighbourhood usually costs, the Committee of the National Society for Employment of Epileptics, which first founded the colony, and of which H.R.H. the Prince of Wales is President, thought it wise to purchase the farm, even though the purchase involved the borrowing of all but £500 of the money. Moreover, the demand for admittance to the colony is greater than can be met. At present female candidates for admittance have to wait for more than two years before they have a chance of acceptance, and males not much less. To meet the requirements of these sufferers it is desirable that two new homes should be built, one for men and one for women, each to take 24 inmates. This would provide for adults, but there are also epileptic children to be considered, in whose case the open-air life, suitable feeding, and well-planned work that characterise the colony, would be invaluable in bringing them up to be as useful as their condition allows, and in the more hopeful cases bringing them approximately near normal health. But for these there is at present no provision. The Society then wants £4,500 to pay off the debt on the newly purchased land, £7,000 to build two new homes for adults, and £8,000 to build two homes and the necessary school accommodation for children. This may seem a large demand, but great also is the good that will be done by the money. The royal President of the Society has expressed his approval of the appeal which is now being made, and which is signed by, among others, the Dukes of

Devonshire and Northumberland, Adeline Duchess of Bedford, the Bishop of Oxford, Lord and Lady Rothschild, and Mr. Passmore Edwards. Subscriptions may be sent either to the Hon. Treasurer, H. N. Hamilton Hoare, Esq., or to the Secretary, G. Penn Gaskell, Esq., at the offices of the Society, Denison House, Vauxhall Bridge Road, Westminster, S.W.

